# Posttranscriptional Regulation of Insulin Resistance: Implications for Metabolic Diseases

**DOI:** 10.3390/biom12020208

**Published:** 2022-01-26

**Authors:** Ana Pérez-García, Marta Torrecilla-Parra, Mario Fernández-de Frutos, Yolanda Martín-Martín, Virginia Pardo-Marqués, Cristina M. Ramírez

**Affiliations:** IMDEA Research Institute of Food and Health Sciences, 28049 Madrid, Spain; ana.perez@imdea.org (A.P.-G.); marta.torrecilla@imdea.org (M.T.-P.); mario.fernandez@imdea.org (M.F.-d.F.); yolanda.martin@imdea.org (Y.M.-M.); virginia.pardo@imdea.org (V.P.-M.)

**Keywords:** insulin resistance, posttranscriptional regulation, miRNAs, RBPs

## Abstract

Insulin resistance defines an impairment in the biologic response to insulin action in target tissues, primarily the liver, muscle, adipose tissue, and brain. Insulin resistance affects physiology in many ways, causing hyperglycemia, hypertension, dyslipidemia, visceral adiposity, hyperinsulinemia, elevated inflammatory markers, and endothelial dysfunction, and its persistence leads to the development metabolic disease, including diabetes, obesity, cardiovascular disease, or nonalcoholic fatty liver disease (NAFLD), as well as neurological disorders such as Alzheimer’s disease. In addition to classical transcriptional factors, posttranscriptional control of gene expression exerted by microRNAs and RNA-binding proteins constitutes a new level of regulation with important implications in metabolic homeostasis. In this review, we describe miRNAs and RBPs that control key genes involved in the insulin signaling pathway and related regulatory networks, and their impact on human metabolic diseases at the molecular level, as well as their potential use for diagnosis and future therapeutics.

## 1. The Insulin Signaling Pathway

**Figure 1:**
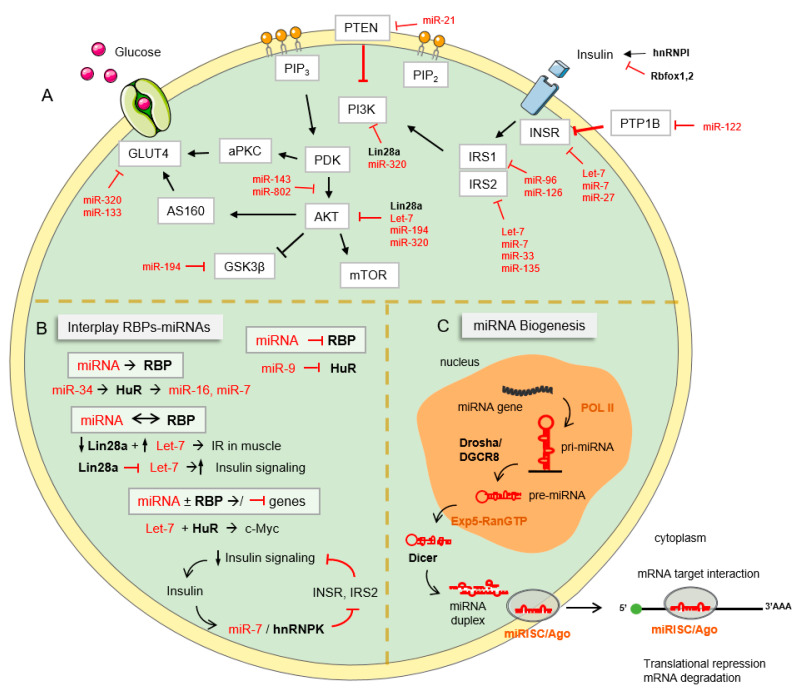
miRNA and RBP regulation of insulin signaling in metabolic diseases. (**A**) Schematic overview of miRNAs and RBPs involved in the regulation of the insulin signaling pathway. (**B**) Cooperation between RBPs and miRNAs. (**C**) General view of microRNA biogenesis. RBPs are represented in bold and miRNAs are represented in red. Black arrows represent activation processes and red lines represent inhibition processes. Note that the target genes showed in the figure are those validated experimentally, but these genes can be also modulated by other miRNAs and the miRNAs highlighted can regulate other genes that do not appear in the figure.

The insulin signaling pathway is a biochemical route which is traditionally known as crucial in the maintenance of glucose homeostasis, being influenced by a wide variety of stimuli such as fasting and feeding conditions, stress levels, and several related hormones. The signaling cascade starts with the binding of the hormone insulin, produced and secreted by pancreatic β cells (β-cells) in response to elevated plasma glucose levels, to the insulin receptor (INSR) on target cells. This binding triggers the activation of several downstream elements by phosphorylation, leading to the transport of glucose, amino acids, and fatty acids, and the conversion of these nutrients into storage macromolecules, such as glycogen, proteins, and lipids [1]. The INSR is an extracellular receptor with tyrosine kinase activity that autophosphorylates after insulin binding, which triggers the activation of the insulin receptor substrate (IRS) and the downstream hub, including the phosphatidyl inositol 3 kinase (PI3K) and protein kinase B (PKB/AKT) pathways. These intracellular signaling cascades promote the activation of glucose metabolism, translocation of glucose transporters to the plasma membrane (GLUT2 in the liver and GLUT4 in other tissues), and synthesis of fatty acids, proteins, and glycogen [2]. IRS proteins also promote cell survival, cell growth and differentiation through the MAP3K pathway [3]. Another critical downstream substrates of AKT are mammalian target of rapamycin (mTOR), glycogen synthase kinase 3 (GSK3) and forkhead box-containing protein, O subfamily (FoxO) transcription factors, especially FoxO1, involved in the regulation of protein synthesis, glycogen synthesis, and gluconeogenic and adipogenic genes, respectively [4]. (Figure 1).

Due to the complexity of the pathway, the crosstalk with other ligands such as insulin-like growth factor (IGF1 and 2) with many downstream elements, and the specificity in different tissues, the mechanisms of regulation and inhibition of the insulin signaling pathway must be extremely controlled to avoid metabolic diseases.

## 2. Insulin Resistance

### 2.1. Definition and Causes of Insulin Resistance (IR)

Insulin resistance defines an impairment in the biologic response to insulin stimulation of target tissues, primarily the liver, muscle, adipose tissue, and brain. The inability to carry out glucose disposal results in an increase in β-cell insulin production and hyperinsulinemia. IR affects physiology in many ways, causing hyperglycemia, hypertension, dyslipidemia, visceral adiposity, hyperuricemia, elevated inflammatory markers, and endothelial dysfunction. If it persists on time, it can lead to the development of metabolic syndrome, NAFLD, cardiovascular diseases (CVD), Alzheimer’s disease (AD) and, most commonly, when β-cells cannot keep up and insulin production gradually decreases until it stops, type 2 diabetes mellitus (T2DM) [5]. Although it has been proven that inflammation has an important role in the development of IR [6], a recent study has shown that insulin resistance precedes and causes inflammation in adipose tissue [7].

Interestingly, nutrients such as resveratrol, purple plant-derived anthocyanin extracts, curcumin, or flavonoids have a relevant role in the improvement of the insulin resistance at molecular levels [8,9,10].

### 2.2. Negative Regulation of Insulin Signaling

Insulin signaling must be tightly controlled so that it does not get overwhelmed, and the activity of the downstream pathways does not cause tumorigenesis or severe disturbances in metabolism derived by IR. Therefore, negative feedback loops at different levels are critical to fine tuning of the network (Figure 1). Some of these inhibitory mechanisms can be altered in pathophysiological conditions, and can also induce the development of IR, diabetes, and related pathologies (discussed later). INSR and IRS proteins are negatively regulated [11] by ligand-induced downregulation by receptor endocytosis coupled with recycling or lysosomal degradation [12,13], by Ser/Thr phosphorylation, and by phosphatases. Even though receptor endocytosis is the main negative regulator, Ser/Thr phosphorylation in response to insulin and other stimuli, including cytokines, free fatty acids, hyperglycemia, and subsequently oxidative stress, as well as mitochondrial dysfunction and endoplasmic reticulum (ER) stress, are also critical factors playing a role in the pathogenesis of insulin resistance [14]. Other associated causes comprise reduced INS quantity or function, reduced INSR availability, mutations in the INRS gene and other elements of the signaling pathway (such as AKT or IRS1), and, less commonly discussed, alterations in the zinc metabolism (Table 1). The phosphorylation of Ser307 of IRS1, for instance, has been correlated with negative regulation of insulin signaling, and it happens to be increased in obese and diabetic mice [15]. Another mechanism of negative regulation of the pathway is the protein and phospholipid phosphatases. Protein Tyrosine Phosphatase 1B (PTP1B) in the ER represents the major and most studied protein tyrosine phosphatase deactivating the INSR [16]. Phospholipid phosphatases such as Phosphatase and Tensin homologue (PTEN) also negatively regulate the activity of the PI3K pathway, interrupting the cascade, avoiding the formation of Phosphatidylinositol-3,4-5,triphosphate (PIP3), thus blocking the cascade since there are no binding sites for AKT [17]. More recently, some novel mechanisms of fine tuning of insulin signaling have been attributed to small non-coding RNAs and RNA binding proteins (RBPs), due to their capacity to control the expression of key elements at the posttranscriptional level [18,19].

## 3. MiRNAs and RBPs: Novel Regulators of Insulin Signaling and Metabolism

It is well established that regulation of metabolism can occur via alternative ways to transcriptional regulation. RBPs and microRNAs are considered main players of posttranscriptional regulation, which controls gene expression via alternative splicing, alternative polyadenylation, RNA editing, alternative translational initiation, inhibition of translation, or mRNA degradation [68,69]. The regulation of these processes occurs by the recognition of the cis-sequences of the mRNAs by RBPs and miRNAs. RBPs and miRNAs can interact with target transcripts at the 3′ and 5′ untranslated regions (UTR), recruit them, and lead to their degradation or to the repression or activation of their translation. Over the past 15 years, important discoveries of several miRNA and RBPs have shown the contribution of posttranscriptional regulation on metabolic dysregulated states and diseases related with insulin resistance, including diabetes, Alzheimer’s disease, and cardiovascular disorders [70,71,72]. One of the most common underlying pathological processes is related to the alteration of gene and protein expression that leads to the impairment of insulin sensitivity and glucose homeostasis, and a vast number of reports has shown altered patterns of miRNAs and RBPs expression during metabolic diseases. All of this raises the possibility of using these molecules as biomarkers for diagnosis or as potential targets for future therapeutics.

### 3.1. miRNAs

MiRNAs (miRs) are a class of small non-coding RNAs measuring 18–25 nucleotides in length that regulate gene expression at the posttranscriptional level and have an important role in many basic biological functions such as cell growth, differentiation, development, and apoptosis [73,74]. The first miRNA, Lin-4, was discovered in 1993 by Lee and colleagues in *C. elegans* [75]. This miRNA is key in postembryonic development, and, intriguingly, it has been shown to interfere with life span through the regulation of insulin/IGF signaling pathway [76,77].

Canonical pathway for miRNA biogenesis implies that miRs are transcribed by polymerase II (PolII) to produce the pri-miRNA that is later processed by the endonuclease Drosha/DGCR8 complex, resulting in the formation of the hairpin precursor (pre-miRNA) that goes under a second cleavage by the endonuclease Dicer and generates a miRNA duplex. One of the strains, the mature ~22 nt (guide strain) is later loaded into the RISC (RNA induced silencing complex) in association with Ago proteins. Through imperfect base-pairing to the 3′ UTR of its target mRNAs, miRNAs regulate the expression of its target genes by repressing mRNA translation and/or promoting mRNA degradation (Figure 1C) [73,78]. Since the discovery of the first miRNA, the posttranscriptional regulation of metabolism by miRNAs has gained significant interest in the last decade, given the number of reports pinpointing their role regulating many intracellular pathways and related cellular functions, including insulin signaling and insulin secretion and production.

### 3.2. RBPs

RBPs are a heterogeneous group of proteins with well-defined RNA binding domains (RBDs) that form ribonucleoprotein (RNP) complexes with RNA. The regulatory effect due to its association with specific RNAs defines transcripts fate by controlling RNA at multiple levels [79]. RBPs can influence all aspects of RNA biology, from transcription, RNA processing, alternative splicing, mRNA stability, and mRNA localization to translation and RNA degradation [68,69]. In addition to the presence of specific sequences that allow the localization and binding of a given RPB to the correct target [80], RNA structure is another decisive variable responsible for the recognition of specific transcripts. The vast number of RNA-sequence specificities and affinities, along with the large diversity of RNA structures, explains why RBPs are involved in a vast variety of cellular processes. Here, we will define well-known RBPs that contribute to several pathogenic events triggered in IR and associated metabolic diseases:

#### 3.2.1. hnRNP Family

The heterogeneous nuclear ribonucleoproteins (hnRNPs) are a set of primarily nuclear proteins that bind to nascent transcripts produced by RNA polymerase II. They regulate gene expression by controlling the maturation of newly formed heterogeneous nuclear RNAs into mRNAs and the stabilization of mRNA during their cellular transport. A collection of 20 major types of hnRNP A-U has been described [81]. The functions of hnRNPs vary according to their cellular localization. On their steady state, they are predominantly nuclear due to the presence in their structure of a conventional nuclear localization signal (NLS). Changes in their subcellular localization from the nucleus to the cytosol are determined by post-translational modification, such as phosphorylation or through the recruitment of other hnRNPs [82]. Thus, hnRNPs such as hnRNPK act in several compartments like the mitochondria in response to insulin, where it binds to mitochondrial DNA and modulates insulin-activated mitochondrial gene expression [83]. 

#### 3.2.2. Hu Family

Hu proteins, identified as target antigens of paraneoplastic neurological syndrome, have been shown to contribute to an ever-growing list of biological functions. Since they share homology with the Drosophila ELAV protein (embryonic lethal abnormal vision), they are typically referred to as the ELAV family. Hu proteins recognize and bind to AU-rich RNA elements (AREs) and other RNA sequences, such as U-rich motifs, in both the 3′UTR and 5′UTR. A total of three of the four Hu members—HuB, HuC and HuD—are predominantly expressed in neurons, where they play essential roles in neuronal differentiation and plasticity, while the ubiquitously expressed family member, HuR, has been found to mostly stabilize the expression of mRNAs that encode proteins that are involved in physiological processes such as adipogenesis, muscle differentiation, and stress and immune responses [84,85]. In addition, HuR has been implicated in cancer, where its expression is predominantly cytoplasmatic, which allows HuR to stabilize and increase the translation of various pro-survival mRNAs involved in tumorigenesis [86]. In relation to other pathologies with metabolic implications such as Alzheimer’s disease, type 2 diabetes, and atherosclerosis, HuR has been also found to be involved in lipid regulation and cholesterol homeostasis [87].

#### 3.2.3. NOVA Family

Neuro-oncologic ventral antigen (NOVA) proteins are splicing factors that regulate neuronal pre-messenger RNA splicing [88,89,90]. The RNA binding properties of the KH domain of NOVA proteins present a considerable medical interest since they have been implicated in a neurodegenerative syndrome known as paraneoplastic opsoclonus-myoclonus ataxia (POMA) [91]. Initially, NOVA proteins were described to be neuro-specific, where the two forms—called NOVA-1 and NOVA-2—carried out different roles. NOVA-1 expression is restricted to neurons in the hindbrain and spinal cord, whereas NOVA-2 can be found in other areas of the central nervous system (CNS), such as the forebrain and thalamus [92,93]. However, more recently it has been reported that NOVA-1 is also expressed in the β-cells in humans and islets of Langerhans in rat [94,95], where it plays key roles in insulin release and β-cell survival through its splicing regulator capacity. 

#### 3.2.4. Rbfox Family

The Rbfox family of splicing factors regulates alternative splicing during animal development and in disease, impacting thousands of exons in the maturing brain, heart, and muscle. Rbfox proteins have a single RNA recognition motif (RRM), which targets with high affinity the RNA sequence GCAUG in pre-mRNA introns, mRNA 3′UTRs, and pre-miRNAs hairpin structures. In vivo, not all Rbfox binding sites contain a (U)GCAUG. Accordingly, Begg et al. described an abundant set of secondary motifs that contributes to Rbfox-dependent gene regulation in vivo and in vitro. Rbfox proteins are predominantly nuclear, but some isoforms are also expressed in the cytoplasm, where they regulate RNA stability [96]. The three Rbfox paralogs—Rbfox1 (A2BP1), Rbfox2 (RBM9), and Rbfox3 (NeuN)—are highly expressed in the heart, and in metabolic active tissues such as the skeletal muscle and the brain [96]. 

#### 3.2.5. CUG-BP Elav-like Family (CELF)

Other class of RBPs that has been strongly linked to alternative splicing are the human family of CUGBP, ELAV-like family (CELF) proteins. CELF is composed by six members classified into two subfamilies: the CELF1-2 subfamily is broadly expressed, while the CELF3-6 subfamily exhibits more restricted expression [97]. Each CELF protein contains three RNA binding domains of the RRM variety, which allow the interaction with certain RNAs [97]. CELF proteins are found both in the nucleus and cytoplasm, where they regulate multiple facets of gene expression in addition to alternative splicing, such as RNA editing, deadenylation, mRNA stability, and translation in various cell types [98]. In vivo, CELF proteins have been shown to play roles in gametogenesis and early embryonic development, heart, skeletal muscle function, and neurosynaptic transmission. Dysregulation of CELF-mediated programs has been implicated in the pathogenesis of human diseases affecting the heart, skeletal muscles, and nervous system [98].

#### 3.2.6. FTO Protein

The fat mass and obesity-associated protein (FTO) belongs to the AlkB family of Fe+/2-oxoglutarate-dependent oxidative DNA/RNA demethylases. Importantly, FTO catalyses the N6-methyl-adenosine (m6A) demethylation in α-ketoglutarate and Fe2+-dependent manners [99]. In this sense, FTO has been consistently associated with human adiposity and metabolic disorders [100,101]. Moreover, FTO targets and reduces the mRNA levels of tuberous sclerosis complex (TSC1), a mTOR upstream inhibitor. Indeed, FTO is also involved in AD by promoting the phosphorylation of Tau in a mTOR-dependent manner. Not surprisingly, brain tissues of diabetic and obese animal models present upregulated levels of FTO, whereas FTO knockdown reduces the phosphorylation of Tau protein [102].

#### 3.2.7. LIN28 Protein

LIN28 is a small protein implicated in RNA binding which is specifically expressed in embryonic cells. In humans, there are two paralogs, LIN28A and LIN28B, which are implicated in pluripotency, reprogramming, and oncogenesis [103,104,105]. LIN28 is a key repressor of Let-7 miRNA biogenesis, a tumor suppressor miRNA, which targets numerous metabolic genes, including those that are involved in the insulin-PI3K-mTOR pathway, such as INSR, IRS2, and IGF1R [106]. LIN28A/B transgenic animals show enhanced insulin sensitivity and lower peripheral glucose levels. In addition, LIN28 overexpression models show a protective effect against T2DM development through let-7 inhibition [106,107].

#### 3.2.8. Dead Box Helicase Superfamily 

DEAD-box (DDX) genes encode a family of RNA helicases that are highly conserved and ubiquitously expressed and appear to participate in almost every aspect of RNA metabolism, including transcription, translation, ribosome biogenesis, RNA splicing, RNA export, and RNA decay. For example, DDX2A (eIF4A1), DDX2B (eIF4A2), DDX3, DDX4, and DDX6 are implicated in translational regulation, DDX5 (p68) and DDX17 (p72) are required for splicing, while DDX21 is involved in ribosomal RNA processing, and DDX23 is a spliceosome component [108]. Regarding their role in metabolism, DDX1 can directly bind to insulin mRNA and stimulate its translation [109]. In vivo, mice treatment with palmitate phosphorylates DDX1 and displaces it from the pre-proinsulin mRNA, suppressing insulin biosynthesis and indicating a direct link between hyperlipidemia and insulin deficiency [109].

Overall, dysregulation of RBPs has been observed in a plethora of diseases, including cancer, as well as neurological, cardiovascular, and metabolic diseases [70,71,72]; what is not surprising given the extensive role of RBPs in the control of gene expression and biological functions. In this sense, RBPs are primed to be the next frontier to explain many of the poorly understood molecular processes dysregulated during metabolic diseases and clarifying the role of diverse RBPs in different physiologic and pathologic settings will highlight novel targets for therapeutic intervention.

### 3.3. Cooperation between RBPs and miRNAs

Although the role of RBPs and miRNAs in regulating gene expression at the posttranscriptional level is indubitable, they were once considered to function independently to regulate the stability and translation of their mRNA targets. However, a growing number of studies suggests a functional interplay between both [110]. Indeed, RBPs can influence the biogenesis of the miRNAs by interacting with a specific pri/pre-miRNA to facilitate or inhibit miRNA processing [111,112]. However, there is more evidence recalling the impact of RBPs in the activity of miRNAs by interfering in their degradation or modifying the mature miRNA form [112,113]. In contrast to the belief that Argonaute (AGO) family proteins was the unique family of RBPs that can interact with mature miRNAs, a number of studies have shown the capacity of different types of RBPs to bind to miRNAs [114]. In this sense, the interaction between miRNAs and RBPs can enhance or antagonize each other’s effects [112,115]. Cooperation with synergistic effects has been described when the repression of a common mRNA occurs when RBPs favor the binding of miRNAs to 3′UTR of target mRNA by modifying its structure. For instance, Gorospe´s laboratory described a synergistic effect between let-7 and HuR in the regulation of c-Myc expression [116]. In contrast, antagonistic effects are observed when a given miRNA and a RBP compete for their binding to the same 3´UTR. A characteristic example of antagonist effects is the interaction between miR-9/HuR. Jeyebal et al. reported the increase of HuR due to repression of miR-9 under hyperglycemic conditions [117]. Other miRs involved in metabolism such as miR-34 are also known to regulate the expression of HuR post-transcriptionally. Conversely, HuR can also regulate the levels of several miRNAs, including miR-16 or miR-7 [118]. Therefore, the levels of RBPs and miRNAs can be reciprocally regulated (Figure 1B). 

Over the last 10 years, significant progress has been made in understanding the role of RBPs and miRNAs in metabolically active and insulin-sensitive tissues. Among the most characteristic examples is the role of Lin28/Let-7 pathway in the regulation of glucose metabolism. Muscle-specific loss of Lin28a and overexpression of let-7 results in IR and impaired glucose tolerance. Furthermore, in vitro, Lin28 increases insulin-PI3K-mTOR signaling due to repression of direct let-7 target in this pathway [106]. These results highlight the potential importance of Lin28/Let-7 during obesity and diabetes. In the same line, we have recently described that miR-7, an intronic miRNA located in the RBP hnRNPK, is co-transcribed with its host gene in response to insulin and it regulates insulin signaling in mouse and human neuronal cells, thus suggesting a possible negative feedback loop of cooperation between them (Figure 1A) [119]. 

## 4. Posttranscriptional Regulation in Metabolic Diseases

The role of many RBPs and miRNAs in insulin production and glucose homeostasis, along with their involvement in insulin signaling pathway, has been increasingly recognized, and that, together with their association with IR and associated diseases, pinpoints posttranscriptional regulators as potential diagnostic tools and targets for future therapies (Table 2) [120,121,122].

### 4.1. Diabetes

Diabetes is one of the main metabolic diseases, characterized by elevated blood sugar levels that lead to serious damage to many tissues over time. According to the World Health Organization (WHO), approximately 422 million people worldwide have diabetes, and 1.6 million deaths are directly attributed to diabetes each year, numbers that have increased at an alarming rate over the past few decades. This prevalence makes it necessary to further study this disease to find new therapeutic targets and strategies. One of the key aspects of diabetes is IR, particularly in T2DM. T2DM occurs when tissue insensitivity to insulin is coupled with an inadequate secretion of insulin by the pancreas [151]. Sustained IR in multiple tissues (including the β-cells) may contribute to loss of insulin secretion, while altered insulin secretion may also increase glucose levels and induce further IR. The burden of IR and T2DM is closely linked to obesity and physical inactivity [152], as both, along with increased consumption of calorie-dense foods and beverages, physiological stressors, systemic inflammation, and oxidative stress, are typically associated with IR. In this line, RBPs and miRNAs have been shown to contribute to pathogenesis of diabetes mellitus and its complications, and hundreds of circulating miRNA have a potential use as biomarkers to evaluate health status and disease progression [153]. Some RBPs and miRNAs have been shown to be implicated in biosynthesis and secretion of insulin and β-cell differentiation, whereas others have a major impact on the insulin signaling pathway [120,121,154]. With respect to the biosynthesis and secretion of insulin, PTBP1, also named as heterogenous ribonucleoprotein I (hnRNPI), has been shown to bind mRNAs encoding preproinsulin and secretory granule proteins and to enhance the insulin secretory capacity of β-cells [123,124,125]. Other RBPs involved in biosynthesis of insulin are HuD, whose expression in β-cells prevents insulin transcription, and DEAD box helicase (DDX1) that influences insulin translation [109,155,156]. NOVA2, Rbfox1 and Rbfox2 are also representative in the pancreas and contribute to β-cells apoptosis and insulin content and secretion, while CUGBP also impairs glucose-stimulated insulin secretion through suppressing intracellular cAMP levels [94,157]. 

In addition to these RBPs, a large suite of miRNAs has been implicated in the function and biology of β-cells. For instance, miR-375 has been shown to play an essential role in β-cells development, regulating a cluster of genes involved in growth-promoting pathways, such as Caveolin-1 [126]. Another miRNA related with β-cells is miR-124, which targets FoxoA2, an important transcription factor in glucose metabolism, insulin secretion, and β-cells differentiation [127,128]. Ramachandran et al. have also shown that miR-9 reduces the glucose-stimulated insulin secretion by targeting Sirtuin 1 (Sirt1) expression, suggesting the important interplay between miR-9, Sirt1 and insulin in diabetes [129]. Interestingly, another miRNA implicated in the insulin biosynthesis is miR-15a. In β-cells, this miRNA targets and inhibits uncoupling protein 2 (UCP2), a negative regulator of insulin secretion, thus overexpression of miR-15a promotes insulin production [130].

On the other hand, RBPs and miRNAs implicated in diabetes have been shown to affect the expression of key components of insulin pathway, such as INSR and IRS. A characteristic example of the role of RBPs/miRNAs in IR and diabetes is the Lin28/Let-7 pathway, mentioned before. In mice models, muscle loss of Lin28 and overexpression of Let-7 resulted in IR and impaired glucose tolerance, whereas, in vitro, Lin-28a improved the glucose uptake, in part due to its inhibition of multiple targets of let-7 in the insulin pathway, such as IGFR, INSR, IRS2, or AKT [104,106]. Several other miRNAs have been described to be involved in IR by regulating INSR and IRSs. These include miR-96 and miR-126, which target IRS1, and miR-33, miR-7, and miR-27, which target INSR and IRS2 [131,132,133,134]. Indeed, miR-135, which was found to be elevated in human diabetic skeletal muscle, also negatively regulates IRS2 expression and impairs phosphorylation of PI3K/AKT by insulin [135]. In other metabolic tissues, such as the liver, the most abundant miRNA, miR-122, inhibits PTP1B, which dephosphorylate INSR and IRS [136]. Other miRNAs have a greater effect on the INSR expression. For instance, Trajkovski et al. demonstrated that miR-103/miR-107 are implicated in the regulation of insulin sensitivity by targeting Cav-1, an important regulator of INSR. In adipocytes, inactivation of these miRNAs leads to the upregulation of Cav-1, stabilization of INSR and enhanced insulin signaling [137]. Finally, several studies involve miRNAs with a key protein in insulin pathway, AKT. MiR-375 is implicated in insulin secretion through the downregulation of the expression of its target PDK1 [158], therefore it attenuates insulin-induced phosphorylation of AKT and GSK3. Interestingly, miR-7 has similar functions than miR-375 [119,159], and both are also highly expressed in the brain. miR-194 also inhibits phosphorylation of AKT and GSK3β, although there is no evidence of direct binding of this miRNA to their 3´-UTR [138].

Recent advances in the field have been focused on decipher the potential use of miRNAs as biomarkers in diabetes, which may help to diagnose disease and avoid severe complications. Patients with T1DM and T2DM show dysregulated miRNA profile in different tissues, which enforces the idea that miRNAs have a significant role during diabetes [160,161]. In addition, several studies have emerged demonstrating different plasma miRNA profiles between diabetic patients and control healthy subject. For instance, miR-15a, miR-29b, miR-126, and miR-223 levels have been found to be lower in subjects with prediabetes or T2DM, and they may be useful to predict the development of diabetes [162]. Several other studies have shown upregulated levels of miRNA in the serum of patients with T2DM [163,164]. Overall, these examples pinpoint the potential impact of miRNAs and RBPs on different pathological processes behind in the development of diabetes.

### 4.2. Obesity

According to WHO, obesity is defined by an abnormal or excessive fat accumulation that represents a risk to health. A plethora of possible harmful states can be caused by obesity, from cardiovascular disease to cancer. Here, we will focus on how this pathological process is regulated post-transcriptionally and its contribution to the development of IR and T2DM.

There is a number of studies showing miRNAs and RBPs associated to obesity. A good example is HuR, which has been shown to protect from diet-induced obesity and IR through the positive regulation of lipolysis [139]. HuR was also identified as an adipogenesis repressor by modulating adipocyte transcripts such as insulin-induce gene 1 protein (Insig1). Correlating with this, levels of HuR are downregulated specifically in the adipose tissue of obese individuals [139]. FTO, another RBP with clear influence in obesity [165], targets the mTOR inhibitor TSC1 [102]. There is a clear association between FTO single nucleotide polymorphisms (SNPs), body mass index (BMI), and the risk of being overweight or obese. This could be explained by the demethylation activity of FTO that might lead to the dysregulation of genes involved in metabolism [166]. Moreover, FTO null mice have a 30–40% weight reduction compared to wild type littermates [167]. Interestingly, the expression of FTO mRNA is very abundant in mouse and human hypothalamus, which contains the neuronal circuity involved in the control appetite [99].

On the other hand, several miRNAs have been found to be upregulated in tissues from obese mice with different outcomes related to IR. Moreover, as it occurs in diabetes, miRNAs might represent functional biomarkers for obesity. MiR-143 and miR-802, for example, increased in the liver of obese mouse models, impair or boost the activation of PKB/AKT by insulin, respectively, exerting opposite effects on IR. miR-143 also regulates adipocyte differentiation and reduces adipogenesis. Indeed, mice deficient for the miR-143 are protected from obesity-associated IR [140,141,168]. Interestingly, several miRNAs involved in IR have a clear impact on adipogenesis. This is the case of miR-103/miR-107, first known for their important role in insulin sensitivity in the liver. These miRNAs were shown to accelerate adipogenesis [169]. Other examples include miR-27b and miR-33, which are known to affect hepatic insulin signaling and inhibit adipogenesis. MiR-27 is a negative regulator of adipocyte differentiation by blocking peroxisome proliferator-activated receptor gamma (PPARγ) and CCAAT-enhancer-binding protein α (C/EBPα) and targets Prohibitin [170,171], whereas miR-33b, encoded in the intron 16 of sterol regulatory element-binding protein 1 (SREBP1) gene in humans, also impairs adipogenesis by targeting of high-mobility group AT-hook (HMGA2) [172]. In adipocytes, miR-320 has been also pinpointed as a relevant regulator of IR. Indeed, inhibition of miR-320 improves insulin sensitivity due to the increase of PI3K-p85α subunit, Glut-4 expression, and AKT phosphorylation [142]. Interestingly, in the liver and in adipose tissue, miR-21 targets PTEN, a negative regulator of the insulin pathway. H.-Y. Ling et al. have demonstrated that this miRNA is downregulated in IR-adipocytes. Other important studies in mice fed with high fat diet and obese patients show that miR-21 expression inversely correlates with PTEN [143,144], highlighting miR-21 as an important therapeutic target in obesity.

In addition to the role of miRNAs in the insulin pathway, several studies demonstrate the link between circulating miRNAs and obesity in human and murine models, suggesting their potential use as biomarkers. These studies primarily show differential levels of circulating miRNA in obese subjects compared to healthy individuals [173,174]. MiR-15-5p and miR-132 are examples of decreased circulating miRNAs in obese versus control subjects [175]. In contrast, miR-222, miR-142–3p, miR-140-5p, and miR-143 are upregulated circulating miRNAs specifically in children with obesity. Indeed, several miRNAs were also dysregulated in children with obesity and IR such as miR-122-5p, miR-34-5p, and miR-320a [176]. Given that miRNAs can facilitate the intercellular communication, they might be relevant as vehicles for metabolic organ crosstalk [173]. However, one of the main questions to answer, together with the targeting of the dysregulated circulating miRNAs in each pathological condition, is the specific tissue where these miRNAs are originated from. Nevertheless, tissue specific knockout models can provide some clues about the origin of circulating miRNA. In this regard, Thomou et al. have shown that the transplantation of adipose tissue from WT mice to a DicerKO mice was sufficient to reconstitute the levels of circulating miRNAs [177].

### 4.3. Cardiovascular Disease

Cardiovascular disease (CVD) is the leading cause of death worldwide. Many of the risk factors associated to CVD, such as hypertension, obesity, and endothelial dysfunction, among others, are linked to IR [178]. Indeed, T2DM is an important risk factor for atherosclerosis, and most diabetic patients die of cardiovascular disease. During IR, several metabolic alterations induce the development of CVD, including imbalances in glucose metabolism, which trigger oxidative stress and cause an inflammatory response, and it can also alter lipid metabolism, which leads to the appearance of low levels of HDL and high levels of triglycerides [178,179]. Atherosclerosis is the main underlying cause of CVD. Pathologically, IR can promote atherogenesis and advance plaque progression at cellular levels, targeting endothelial cells, vascular smooth muscle cells, and macrophages; all of the above express INSR and insulin receptor-mediated signaling pathway elements. On the other hand, there is evidence that suggests that insulin is an atherogenic hormone. It has been shown to increase formation of lipid plaques, cause proliferation of smooth muscle cells, increase LDL receptor activity, and stimulate growth factors, among others [180]. Defective pathways such as PI3K-AKT, IRS-1, and MAP kinase may induce endothelial dysfunction by interfering with generation of vasodilatory and vasoconstrictive substances, such as nitric oxide (NO) [179]. Endothelial dysfunction is the main cause of heart failure, and has a close relationship with insulin signaling and IR. Under normal conditions, insulin regulates the production of NO in endothelium to regulate vascular functions, but in an insulin-resistant state, there are imbalances in NO production that can increase cardiovascular risk [181]. More evidence showing the importance of IR in CVD comes from the studies of atherosclerosis in the absence of AKTs [182,183].

In recent years, the role of miRNAs and RBPs in the context of cardiovascular diseases has experienced significant growth. Numerous studies explored control of glucose, lipid metabolism, and IR in the context of diabetes and obesity, but little is known about miRNA and RBPs contributing to IR association with cardiovascular diseases. In this review, we aim to compile the main miRNAs and RBPs involved in the posttranscriptional regulation in CVD related to IR.

MiR-9/HuR represent a good example, since both are implicated in hyperglycemia and pyroptosis in cardiomyocytes [117], suggesting their possible implication in cardiovascular pathologies associated with IR and diabetes. This work demonstrated that miR-9 targets HuR, and that their expression inversely correlates in human diabetic heart. HuR has been also shown to regulate other notorious proteins implicated in many cardiovascular conditions, such as Angiotensin II type 1 receptor (AT1R), which is upregulated under hyperinsulemic situations. In this line, Paukku et al. have demonstrated that HuR is necessary for the stabilization of AT1R mRNA by insulin [184]. Additionally, recent studies have demonstrated the potential role of HuR in atherosclerosis by regulating cholesterol efflux and lipid metabolism in macrophages [87]. Other RBPs such as RBFOX2 can also contribute to complications in diabetic hearts, due to its capacity to control alternative splicing of key genes, such as myotubularin related protein 3 (Mtmr3) or Fragile X mental retardation syndrome-related protein 1 (Fxr1), involved in heart function and cardiovascular disease [185]. Finally, Lin28a, implicated in insulin-PI3K-mTOR pathway, might constitute a potential therapeutic target in diabetic cardiomyopathy, as its levels are impaired in the diabetic heart [105].

In the context of miRNAs, several studies have also highlighted the importance of miRNAs in regulating cardiovascular disease by targeting IR. Some of the described miRNAs include miR-1 and miR-206, which are abundant in cardiac muscle and are known to target IGF-1. In cardiomyocyte cell lines, miR-1 blocked the capacity of IGF-1 to prevent glucose-induced mitochondrial dysfunction and apoptosis [186]. Similarly, Shan et al. provided data indicating that miR-1 and miR-206 regulate Hsp60 and IGF-1 expression, contributing to glucose-mediated apoptosis in cardiomyocytes [187]. These studies show a potential role of both miRNAs in myocardial infarction. Additionally, there are miRNAs that also target IGF-1, such as miR-320, that improves angiogenesis in diabetic rats [188]. Other miRNAs described in the literature known to target additional components of insulin signaling pathway in cardiomyocytes include miR-223 and miR-133. Overexpression of miR-223 increases GLUT4 levels and inhibits insulin-stimulated AKT and GSK3β phosphorylation [145], while the overexpression of miR-133 decreases GLUT-4 levels [146], demonstrating their implication in IR and metabolic control in the heart and during cardiovascular disease. Research evidence from different laboratories has proven that miRNAs that are implicated in CVD are related to endothelial and vascular smooth muscle cell dysfunction, macrophage and platelet activation, lipid metabolism abnormality, and cardiomyocyte repolarization in diabetes [189]. For instance, during hyperglycemia, miR-34a was found to be increased in mouse microvascular endothelial cells, which is accompanied by a decrease in SIRT1 and impaired angiogenesis [190]. MiR-504 levels have been shown to be upregulated in the aortic vascular smooth muscle cells (VSMCs) of diabetic mice, which promotes their proliferation and migration and leads to vascular dysfunction [191]. Finally, hyperglycemia can also cause electrophysiological changes in cardiac progenitor cells (CPCs) and the increase of miR-1/133 in these cells, suggesting their potential implication in arrhythmia [192].

Collectively, these studies show that miRNAs are important players affecting the diverse aspects of diabetic CVD. Although further research is required, miRNAs may be a novel therapeutic approach for alleviating diabetes-induced progression of cardiovascular complications.

### 4.4. Alzheimer’s Disease

Alzheimer’s disease (AD) is the most common form of dementia in elderly people and is among the group of diseases leading causes of death worldwide [193]. AD is a degenerative and progressive disease with ambiguous pathology; however, accumulation of insoluble extracellular amyloid beta (Aβ) plaques and the formation of intracellular neurofibrillary tangles (NFT) are the main factors for AD pathogenesis [194]. AD may be classified as early onset, which is inherited via an autosomal dominant pattern and a second type, or late onset which is a spontaneous form, also known as sporadic AD, linked to metabolic alterations. In this context, epidemiological clues indicate a close relationship between insulin resistance with AD and memory impairment [195,196]. Many recent studies have demonstrated that AD is a degenerative metabolic disease characterized by a defective glucose utilization, brain insulin responsiveness, and energy metabolism, which are risks factors to oxidative stress and neuroinflammation [197,198,199]. Indeed, IR leads to a diabetic state in brain, also known as Type 3 diabetes. In the CNS, insulin, IGF and all of the elements of this signaling pathway play a crucial role in neuronal growth, synaptic maintenance, neuronal stem cell activation, neuroprotection, and Aβ degradation [200]. Furthermore, insulin in the brain regulates glucose and lipid metabolism, and also has a key role in learning and memory [199,201]. There are many excellent reviews about pathophysiology of AD with metabolic alterations, so here, we will mainly focus on the posttranscriptional regulation of two key pathological aspects of AD, the Aβ oligomers and hyperphosphorylation of Tau, and the link with IR. Insulin can modulate the clearance of extracellular Aβ oligomers and its neurotoxic effects by regulating Insulin degrading enzyme (IDE) expression. IDE is secreted by microglial cells to exert its key role in Aβ metabolism, besides its role in insulin catabolism [202]. On the other hand, Tau is a microtubule-associated protein found in axons [202], which acts in the assembly and stability of microtubules and in vesicle transport in neurons. However, the non-soluble hyperphosphorylated form of Tau can produce its aggregation in neuronal axons and NFT, leading to impaired transport along the axons and synaptic loss, among other effects [203,204]. Many studies have shown the link between Aβ production, Tau hyperphosphorylation with defects in IDE, and failure in the inhibition of GSK3-β during IR states [200,205].

The role of miRNAs involved in neurodegeneration has emerged as a major focus of interest in recent years, and growing evidence highlights the relationship between AD and diabetes, and their association with dysregulation of insulin signaling [206]. Importantly, the brain is the organ that expresses the most distinct and largest number of miRNAs than any other tissue [207,208,209]. However, although there are evidence showing the role of posttranscriptional regulatory mechanisms implicated in the pathology of AD, especially by miRNAs, many of these studies had been mainly focused on the modulation of key AD-associated genes such as APP, PS1, etc. [210]. Thus, few studies have explored the potential of targeting miRNAs/RBPS related to IR for the treatment of AD, and further research in this area is needed.

Some of the best known miRNAs in the context of IR in AD include miR-200b/c, miR-98, and miR-7, among others [211], and they have in common their impact on components of insulin signaling pathway. For instance, miR-200b/c reduces S6K1 dependent IRS-1pSer and IR and inhibits Aβ production in the human neuroblastoma cell line SH-SY5Y, contributing to positive outcomes in AD [147]. MiRNAs involved in AD pathology can also interfere with INSR and indirectly affect downstream elements. This is the case of miR-7. In our laboratory, we have demonstrated that miR-7 targets and represses several components of the insulin pathway such as INSR and IRS2, together with important regulators of AD such as IDE. Our work shows that overexpression of miR-7 in neuronal cells increases production of Aβ. In addition, this miRNA is upregulated in brain of AD patients, which inversely correlates with the expression of its target genes IRS2 and IDE. Ongoing studies using AD-mouse models will elucidate the role of miR-7 in the simultaneous regulation of IR and AD in vivo [119]. Likewise, other miRNAs related to neurodegeneration are linked to the posttranscriptional regulation of IGF-1, such as miR-29, miR-98 or miR-7 [212]. In the neuroblastoma cell line N2a, miR-98 targets and downregulates IGF-1 and increases Aβ formation and Tau phosphorylation [148]. MiR-26 also targets and downregulates IGF-1 and enhances Aβ production [149]. IGF-1 is also targeted by miR-29 and elevated levels of this miRNA in the brain of aged mice and in human cortical tissues negatively correlate with levels of IGF-1 in vivo [150].

In a similar fashion as with other human diseases, finding differential patterns of miRNAs profiles comparing AD vs. healthy subjects has been pursued in research, as some of these differentially expressed RNA molecules could represent potential alternative biomarkers for diagnosis and prognosis of AD. There are several studies that show distinct profiles of circulating miRNAs between healthy and AD patients. Schipper et al. found that miR-34a and 181b levels were significantly elevated in the plasma during AD compared to healthy individuals. Moreover, the levels of miR-181b were also higher in ApoE4 positive AD subjects, suggesting that this miRNA can be associated with an important AD genetic risk factor [213]. Other studies have shown differential miRNAs in different regions of the AD brain [214]. For example, in the hippocampus and medial frontal gyrus, important regions affected by AD neurodegeneration, the expression of miR-200c, -212, -26a, etc. was altered in the early and late stages of the disease. Interestingly, specific pro-inflammatory miRNAs, such as miR-9, miRNA-125b, miRNA-146a, and miRNA-155, are abundant in the extracellular fluid (ECF) and cerebrospinal fluid (CSF) of AD patients, and they associate with the progressive spreading of inflammatory neurodegeneration [215,216]. Let7b, a well-known metabolic regulator of gene expression in CNS, is also increased in the CSF of AD subjects. Interestingly, injection of let-7b into the CSF in mice resulted in neurodegeneration [217]. By contrast, other downregulated miRNAs have been found in the serum of AD patients and mouse models, such as miR-137, miR-181c, miR-9, and miR-29a/b [218]. Regarding RBPs, little is known about their functions in the context of IR during AD. In this sense, a recent interesting study has proved that FTO targets and reduces the mRNA levels of mTOR inhibitor TSC1 [102], showing that FTO activates the phosphorylation of Tau in a mTOR-dependent manner. Not surprisingly, brain tissues of diabetic and obese animal models present upregulated levels of FTO, whereas FTO knockdown reduced the phosphorylation of Tau protein, suggesting a possible underlying common mechanism linking IR and the pathology of AD [102]. Overall, to accelerate research in the field of miRNAs as biomarkers in the context AD is especially important, since early diagnosis could provide better care and quality of life for millions of patients and families worldwide.

## 5. Conclusions

Given that metabolic dysregulation underlies many human diseases that represent major public health concerns such as diabetes or AD, the importance of developing alternatives for treating, diagnose and managing metabolic diseases is a high priority matter, especially for those that lack curative therapy. Differential patterns of miRNA expression have been found in tissues and in circulation from animal models and in human subjects suffering from IR-associated states such as obesity, diabetes, cardiovascular, or AD, compared to healthy subjects. Although their potential use for the specific treatment of a single disease needs to be explored more extensively, and miRNAs could be relatively tissue-specific, it is widely accepted that circulating miRNA may have the potential as novel biomarkers. Besides that, modifying the behavior of miRNAs or correcting dysregulated levels during disease, could be used as potential therapeutics for targeting metabolic pathologies [18]. For instance, miRNA antisense oligonucleotides (ASOs), or modified 2′-O-methyl (2′-O-Me), 2′-O-MOE, and locked nucleic acid (LNAs) have been tested to evaluate their potency to inhibit miR-122, miR-103/107, and miR-33 (all involved in IR) in metabolic active tissues such as the liver. In addition to antagomirs, miRNA sponges, binding site protectors or other small molecules might be an alternative approach to suppress miRNA activity, the latter with special importance in brain diseases where the blood-brain barrier (BBB) must be crossed [219]. Although recent advances in the design of therapeutic strategies for miRNAs have been made, it is important to keep in mind that miRNAs can potentially target hundreds of mRNAs, and they likely function by simultaneously targeting sets of mRNAs involved in specific biological functions or cellular routes [220]. To complicate the matter further, miRNAs could work in cooperation with other posttranscriptional elements such as RBPs or long noncoding RNAs, and therein lies their effectiveness, as they can jointly affect expression of many proteins within a given pathway. Finally, it is interesting to note that miRNAs and RBPs go beyond posttranscriptional regulation from the moment they interfere with the expression of many transcription factors and cofactors. The interplay between transcription factors and RBPs or miRNAs and, more importantly, their reciprocal influence, comprise an intricate and complex system for fine tuning gene expression. Therefore, a comprehensive knowledge of the molecular post-transcriptional influence of RBPs and miRNAs in metabolic diseases includes a better understanding of the factors regulating their expression, so as to design effective therapeutic strategies for treatment in the near future [18].

## Figures and Tables

**Table 1:** Etiology of insulin resistance (IR).

Cause	Gene/Protein	Mechanism	Reference
Reduced insulin quantity or function	-	Autoimmune antibodies	[20]
	INS	Mutations in the insulin gene	[21,22]
Reduced INSR availability	-	Reduced exposure in the membrane	[23,24]
	-	Autoimmune antibodies	[25]
INSR mutations	INSR	Accelerated degradation	[26,27]
		In the ligand-binding domain	[28,29]
		In the tyrosine kinase domain	[27,30]
		Reduced mRNA expression	[31]
Mutations in other elements of the pathway	IRS-1	Impaired insulin signaling	[32,33,34,35]
	PTEN	Impaired end of signaling	[36,37]
	GLUT-4	Reduced glucose internalization in target tissues	[38]
	AKT and its targets	Impaired insulin signaling	[39,40,41,42]
Lipotoxicity	Lipoprotein lipase	Overexpression in muscle	[43]
	IKK and JNK	Endoplasmic reticulum stress by circulating FFA or ceramides	[44,45]
	Serine/threonine (Ser/Thr) kinases (PKC-θ, PKCβII and PKCδ)	DAG-induced activation	[46]
	IKKβ and NFκB	DAG-induced inhibition	[47]
Inflammation	Proinflammatory cytokines (MCP-1, TNF-α, IL1β or IL-6)	Adipocytes and macrophages triggered in obesity Inhibition of several steps of the insulin pathway	[15,48,49,50,51]
Mitochondrial Dysfunction	-	Reduced content and/or biogenesis	[52,53]
	-	Decreased ATP production and phosphocreatine recovery	[54,55,56,57]
	Citrate synthase	Decreased activity	[58]
	-	Lower OxPhos capacity	[59]
	-	Increased ROS	[60,61,62]
Alterations in the zinc metabolism	Zinc transporters (ZnT8, SLC30A8, ZnT7)	Regulation of multiple zinc-dependent effectors	[63,64,65,66,67]

The symbol “-” has been used when no specific gene or protein is related to the mechanism described.

**Table 2:** miRNAs and RBPs involved in insulin signaling regulation.

Regulator	Tissue/Cell Type	Target Genes	Function	Disease	Reference
hnRNPI	β-cells	INS	↑ Insulin secretion	T2DM	[123,124,125]
Rbfox1,2	β-cells	INS	↓ Insulin secretion	T2DM	[94]
miR-375	β-cells	CAV-1	β-cell development	T2DM	[126]
miR-124	β-cells	FOXA2	↓ Insulin secretion	T2DM	[127,128]
miR-9	β-cells	SIRT1	↓ Insulin secretion	T2DM	[129]
miR-15a	β-cells	UCP2	↑ Insulin biosynthesis and secretion	T2DM	[130]
Lin28/Let-7	Muscle	IGF1R, INSR, IRS2, AKT	Insulin signaling	T2DM	[104,106]
miR-96, miR-126	Hepatocytes	IRS1	↓ Insulin signaling	T2DM	[131,132]
miR-27, miR-33	Hepatocytes	INSR, IRS2	↓ Insulin signaling	T2DM	[133,134]
miR-135	Skeletal muscle	IRS2, PI3K/AKT	↓ Insulin signaling	T2DM	[135]
miR-122	Liver	PTP1B	↑ Insulin signaling	T2DM	[136]
miR-103, miR-107	Adipocytes	CAV-1	↓Insulin sensitivity	T2DM	[137]
miR-194	Skeletal muscle	AKT, GSK3β	↓Glucose metabolism	T2DM	[138]
HuR	Adipocytes	INSIG1	↑ Insulin sensitivity	Obesity	[139]
miR-143, miR-802	Liver	PKD/AKT	↓ Insulin signaling	Obesity	[140,141]
miR-320	Adipocytes	PI3K, GLUT4, AKT	↓Insulin sensitivity	Obesity	[142]
miR-21	Liver, adipose tissue	PTEN	↑ Insulin signaling	Obesity	[143,144]
Lin28a	Heart	PI3K, AKT	↑ Insulin sensitivity	CVD	[105,106]
miR-223 miR-133	Cardiomyocytes	GLUT4 GLUT4	↑ Glucose uptake ↓ Glucose uptake	CVD CVD	[145] [146]
miR-200b/c	Murine primary neurons	S6K1	↑ Insulin signaling	AD	[147]
miR-7	Neuronal cells	INSR, IRS2, IDE	↓ Insulin signaling	AD	[119]
miR-26, miR-29, miR-98	Neuronal cells	IGF1	↑ Aβ production and Tau phosphorylation	AD	[148,149,150]

“↑” means increase, “↓” means decreased.

## Abbreviations

AD: Alzheimer’s disease; CVD: Cardiovascular Disease; DDX: DEAD-box; FoxO: Forkhead box-containing protein, O subfamily; FTO: Fat Mass and Obesity-associated protein; GLUT- Glucose Transporter; GSK3: Glycogen Synthase Kinase 3; hnRNP: heterogeneous nuclear ribonucleoprotein; IDE: Insulin Degrading Enzyme; IGF: Insulin-like Growth Factor I; INSR: Insulin receptor; IR: Insulin Resistance; IRS: Insulin Receptor Substrate; MiRs: MiRNAs; mTOR: mammalian Target of Rapamycin; NAFLD: Nonalcoholic Fatty Liver Disease; PI3K: Phosphatidyl Inositol 3 Kinase; PKB/AKT: Protein kinase B; PTEN: Phosphatase and Tensin homologue; PTP1B: Protein Tyrosine Phosphatase 1B; RBPs: RNA binding proteins; T2DM: Type 2 Diabetes Mellitus.
